# A biostimulant yeast,* Hanseniaspora opuntiae,* modifies *Arabidopsis thaliana* root architecture and improves the plant defense response against *Botrytis cinerea*

**DOI:** 10.1007/s00425-023-04326-6

**Published:** 2024-01-31

**Authors:** Israel Maruri-López, Yordan J. Romero-Contreras, Selene Napsucialy-Mendivil, Enrique González-Pérez, Norma Yaniri Aviles-Baltazar, Ana Isabel Chávez-Martínez, Everardo Jair Flores-Cuevas, Kátia Regina Freitas Schwan-Estrada, Joseph G. Dubrovsky, Juan Francisco Jiménez-Bremont, Mario Serrano

**Affiliations:** 1 Centro de Ciencias Genómicas, Universidad Nacional Autónoma de México, Cuernavaca, Morelos Mexico; 2 Laboratorio de Biología Molecular de Hongos y Plantas, División de Biología Molecular, Instituto Potosino de Investigación Científca y Tecnológica AC, San Luis Potosí, Mexico; 3 Instituto de Biotecnología, Universidad Nacional Autónoma de México, Cuernavaca, Morelos Mexico; 4 Departamento de Agronomia, Universidade Estadual de Maringá, Maringá, 87020 Brazil; 5 Present Address: Facultad de Ciencias, Universidad Autónoma de San Luis Potosí (UASLP), Av. Chapultepec 1570, Priv. del Pedregal, 78295 San Luis Potosí, Mexico; 6 Present Address: Center for Desert Agriculture, Biological and Environmental Science and Engineering Division (BESE), King Abdullah University of Science and Technology (KAUST), Thuwal, Saudi Arabia

**Keywords:** Auxin and ethylene signaling, Biostimulant, Innate immunity, Plant–yeast interaction, Systemic defense responses, Volatile organic compounds

## Abstract

**Main conclusion:**

The biostimulant *Hanseniaspora opuntiae* regulates *Arabidopsis thaliana* root development and resistance to *Botrytis cinerea*.

**Abstract:**

Beneficial microbes can increase plant nutrient accessibility and uptake, promote abiotic stress tolerance, and enhance disease resistance, while pathogenic microorganisms cause plant disease, affecting cellular homeostasis and leading to cell death in the most critical cases. Commonly, plants use specialized pattern recognition receptors to perceive beneficial or pathogen microorganisms. Although bacteria have been the most studied plant-associated beneficial microbes, the analysis of yeasts is receiving less attention. This study assessed the role of *Hanseniaspora opuntiae,* a fermentative yeast isolated from cacao musts, during *Arabidopsis thaliana* growth, development, and defense response to fungal pathogens. We evaluated the *A. thaliana–H. opuntiae* interaction using direct and indirect in vitro systems. Arabidopsis growth was significantly increased seven days post-inoculation with *H. opuntiae* during indirect interaction. Moreover, we observed that *H. opuntiae* cells had a strong auxin-like effect in *A. thaliana* root development during in vitro interaction. We show that 3-methyl-1-butanol and ethanol are the main volatile compounds produced by *H. opuntiae.* Subsequently, it was determined that *A. thaliana* plants inoculated with *H. opuntiae* have a long-lasting and systemic effect against *Botrytis cinerea* infection, but independently of auxin, ethylene, salicylic acid, or jasmonic acid pathways. Our results demonstrate that *H. opuntiae* is an important biostimulant that acts by regulating plant development and pathogen resistance through different hormone-related responses.

**Supplementary Information:**

The online version contains supplementary material available at 10.1007/s00425-023-04326-6.

## Introduction

During their lifespan, plants interact with multiple microbes, such as bacteria, oomycetes, filamentous fungi, and yeasts. It is well known that microbes can reside in the phyllosphere, endosphere, and rhizosphere regions (Wang et al. 2016; Hawkins and Crawford 2018; Khare et al. 2018; Jones et al. 2019). Some microbes can benefit plant growth and help them survive in changing environments, while others cause many plant diseases (Compant et al. 2010; Dean et al. 2012; Contreras-Cornejo et al. 2014). The most studied beneficial bacteria and filamentous fungi belong to the *Bacillus* and *Trichoderma* genera (Wang et al. 2016; Nieto-Jacobo et al. 2017; Jones et al. 2019; Kang et al. 2019), whereas yeasts have received the least attention. On the other hand, plant-associated pathogens include bacterial and fungal agents, such as *Erwinia, Pseudomonas, Phytophthora,* and *Botrytis,* among other genera (Mansfield et al. 2012; Dean et al. 2012; De Simone et al. 2020; Donati et al. 2020).

When confronted by microbes, plants use a multifaceted defense system to identify foreign organisms and turn the related signals into defense, such as expressing plant defense response genes. Depending on the microbe lifestyle, the plant immune system employs a wide range of different strategies, which include changes in gene transcription, protein regulation, epigenetic modifications, small RNAs biosynthesis, signal molecules, and accumulation of phytohormones, which can induce defense at a local and/or systemic levels throughout plant organs (Bulgarelli et al. 2013; Mikiciński et al. 2016; Alonso et al. 2019; Fiorilli et al. 2020). At the same time, a balance exists between defense response and growth tradeoff (Cai and Aharoni 2022).

*Arabidopsis thaliana* is a well-described model organism for plant–microbe interactions analysis (Dubrovsky et al. 1994). This model has allowed the study of host and non-host pathogen interaction systems, root-associated microbiomes, and endophytic microorganisms (Zimmerli et al. 2004; Contreras-Cornejo et al. 2009: Wang et al. 2016; González-Pérez et al. 2018, 2022; Kudjordjie et al. 2021). Thus, the use of *A. thaliana* has brought to light the basis of plant immunity, such as those mechanisms involved in the perception of microbe-associated molecular patterns (MAMPs) in MAMP-triggered immunity and Resistance (R)-gene function in the signaling of effector-triggered immunity (Jones and Dangl 2006; Li et al. 2020). Although these investigations have centered on bacteria, filamentous fungi, and oomycetes, *A. thaliana* is also used as a model organism to study plant–yeast interactions (Wang et al. 2016). Knowledge of plant–yeast interactions would be helpful for a more comprehensive understanding of scenarios of plant immunity responses related to beneficial microorganisms.

Members of *Hanseniaspora* yeast genus are found in grape, citrus, and cacao musts, which are usually the most abundant microorganisms at the beginning of the fermentation (Ferreira-Saab et al. 2018; Hu et al. 2020; Saubin et al. 2020). *Hanseniaspora opuntiae* was isolated from the *Opuntia ficus-indica* rot (Cadez et al. 2003) and was considered a biocontrol agent against fungal plant pathogens, such as *Corynespora cassiicola* and *Botrytis cinerea* (Ferreira-Saab et al. 2018). Previously, we reported that *A. thaliana* plants treated with compounds released by the biocontrol yeast *H. opuntiae* [*H. opuntiae*-Filtrates (HoFs)] were resistant to the necrotrophic fungus *Botrytis cinerea*. We observed that HoFs induced the plant defense response in a dose-dependent manner at local and systemic levels. Moreover, by performing an RNA-seq analysis, we identified that HoFs triggered different plant defense response pathways compared to other previously described biocontrol agents (Ferreira-Saab et al. 2018).

In this study, we explored the role of *H. opuntiae* in *A. thaliana* growth, development, and defense response to the fungal pathogen *B. cinerea*. We established that *H. opuntiae* had a strong auxin-like effect on *A. thaliana* root development during the direct and indirect in vitro interaction. Afterward, we analyzed the resistance to *B. cinerea* in plants inoculated with *H. opuntiae*. Our results suggest that this yeast induces plant resistance to fungal infection by cooperating with multiple hormonal response pathways. We propose that *H. opuntiae* acts as a biostimulant capable of improving plant growth and controlling fungal pathogens.

## Materials and methods

### Plant material and growth conditions

Seeds of *A. thaliana* Col-0 wild type*, DR5:uidA* auxin reporter line, *rhd6* root hair defective mutant and mutants related to auxin- (*axr2-1 and tir1*), ethylene signaling (*ein3*), salicylic acid (*eds5, npr1-1 and npr3-1*), and jasmonic acid (*jar1*) were used in this study. Seeds were surface sterilized with 70% (v/v) ethanol for five minutes and 96% (v/v) ethanol for five minutes. After ethanol washing, seeds were grown in square Petri dishes on 0.2X Murashige and Skoog (MS) growth medium, pH 5.7, containing 0.5% (*w*/*v*) sucrose and 0.8% (*w*/*v*) agar (Murashige and Skoog 1962). Seeds were stratified for 2 days at 4 ºC in the dark and then the Petri dishes were incubated in a vertical position at 22 ± 2 ºC in a growth chamber with a 16-h photoperiod (120 μmol m^−2^ s^−1^).

### Yeast strain and growth conditions

*Hanseniaspora opuntiae* CCMA 0760 from CCMA (Culture Collection of Agricultural Microbiology, http://www.ccma.dbi.ufla.br/; Universidade Federal de Lavras) was the yeast strain used to perform the experiments. Yeast cells were grown in 0.67% Yeast Nitrogen Base (YNB) supplemented with 2% glucose. Two percent of agar was added to a prepared solid medium. Yeast was grown on YNB plates for 2 days at 28 °C, and the yeast cell suspension was obtained in a YNB liquid medium. The total cell number was calculated in a Neubauer chamber under 40 × magnification in a Zeiss Axioskop 2 microscope using an objective Plan Neofluar 40x/0.75 Ph2.

### *H. opuntiae*–*A. thaliana* interactions

Two co-culture conditions were evaluated: direct and indirect treatments described in the Results section. The yeast inoculation was carried out seven days after germination of *A. thaliana* seeds (Col-0, *DR5:uidA, rhd6 and tir1*) which were inoculated with 20 µL per plate of a suspension of 1 × 10^10^ cell/mL; then plants were analyzed at seven days post-inoculation (dpi). Plantlets without inoculum were used as a control. In the direct treatment experiments, yeast culture was placed at the bottom of the dish five cm from the root tips of *A. thaliana* seedlings (10 seedlings per dish). During an indirect treatment, to avoid direct contact between plantlets and yeast culture, for which the middle segment of agar (∼2 mm thick) was entirely excised from square plates. Five plantlets were grown on the left side of the Petri dish, and the yeast culture was directly placed first from the bottom of the right side of the dish and then spread out through all the agar surfaces of the medium at the right side of the dish. Yeast-treated and untreated control dishes were incubated vertically at 22 ± 2 ºC in a growth chamber with a photoperiod of 16-h light (120 μmol m^−2^ s^−1^).

For pot inoculation assays, 4-week-old *A. thaliana* plants (Col-0, *axr2-1, ein3, eds5, npr1-1, npr3-1,* and *jar1*) were pre-treated by watering with 1 mL of a suspension of 6 × 10^10^ cells/ mL every other day for one week. Yeast cells were grown overnight in 0.67% YNB supplemented with 2% glucose and then were inoculated in 0.2X MS medium with 0.5 sucrose until the desired cell concentration. After this time, five μL droplets of *B. cinerea* spore suspension (5 × 10^4^ spores/mL) were applied to the leaves surface. The parameters evaluated from *B. cinerea* infection, including disease incidence and measurement of lesion size, were determined at three dpi.

### Evaluation of plant morphological parameters

Fresh weight, primary root length, and total chlorophyll content were determined in plantlets seven dpi. Three groups of ten plants were measured for the direct treatment, and three groups of five plantlets were used for the experiments of indirect treatment*.* ImageJ software was used to estimate the primary root length (cm). Plantlet fresh weight (g) was obtained using an analytical scale balance and the value obtained represents the mean of one plantlet (*n* = 30). Whole plants were submerged in 2 mL of 80% ethanol at room temperature in darkness for chlorophyll extraction and quantification. After 2 h, chlorophyll content was determined by measuring absorption spectra at 664 and 647 nm (Hiscox and Israelstam 1979). The concentration of chlorophyll per gram of fresh weight was calculated as follows: μMoles of chlorophyll = 7.93 (A_664_) + 19.53 (A_647_). All experiments were repeated at least three times with similar results.

### Analysis of root development

To evaluate the primary root growth dynamics, we marked every 24 h the position of the root tip over the plates maintained in a vertical position. At the end of the experiment, the Petri dishes were scanned, and the root growth increments were measured using ImageJ software. From these data, the total root length was evaluated. The analysis of lateral root (LR) and LR primordium (LRP) density, length of fully elongated cells, and LR initiation index were determined on cleared roots (Dubrovsky et al. 1994). To clear the roots, we used the Malamy and Benfey protocol (1997) and some modifications described in Dubrovsky et al*.* (1994). The analysis of whole-mount preparations was performed using an Olympus BX53 microscope (Tokyo, Japan) equipped with differential interferential contrast (DIC; Nomarski) optics.

### GUS histochemical analysis of *DR5:uidA* line

We placed seven-day-old *DR5:uidA* seedlings with yeast cells, as previously mentioned. To analyze the effect of auxin synthesis inhibitor, the 0.2X MS growth medium was supplemented with 10 μM 4-phenoxyphenylboronic acid (PPBo). After seven dpi, the *DR5:uidA* seedlings were subjected to GUS histochemical staining during 12 h of incubation at 37 °C in a GUS reaction buffer (0.5 mg/mL of 5-bromo-4-chloro-3-indolyl-β-D-glucuronide in 100 mM sodium phosphate, pH 7) (Jefferson et al. 1987). The tissue clarification process was performed as described by Malamy and Benfey (1997). At least 15 plantlets (five per Petri dish) were analyzed at a 10 × magnification in a Zeiss Axioskop 2 microscope for both types of treatment. All experiments were carried out at least three times with similar results. For the quantification of GUS intensity, the images were processed using the ImageJ software. The images were adjusted to the same resolution intensity, and the color intensity was measured through a threshold analysis.

### Root hair analyses of the *rhd6* mutant

The *A. thaliana* seedlings of *rhd6* mutant were grown for seven days, and then plants were subjected to direct and indirect treatments performed in 0.2X MS and 0.2X MS growth medium supplemented with 50 mM CuSO_4_. The primary root portion from 500 to 1000 μm from the root tip was analyzed at seven dpi under 10 × magnification in a Zeiss Axioskop 2 microscope. Ten plants were analyzed for each treatment (*n* = 10). All experiments were performed at least three times with similar results.

### Collection of Volatile Organic Compounds (VOCs) by SPME and GC − MS analysis

To identify the VOCs produced in *A. thaliana-H. opuntiae* co-cultivation, GC/MS analysis was conducted. *A. thaliana–H. opuntiae* interactions were carried out in a shared atmosphere using Petri dishes with partitioned growth medium. The experimental conditions assessed were as follows: (i) VOCs emitted by *H. opuntiae* growth without *A. thaliana* plantlets and (ii) indirect interactions when *H. opuntiae* was grown on MS medium. Also, as a control, the background of VOCs produced by *A. thaliana* and culture media MS was assessed. VOCs were extracted using a solid phase micro-extraction SPME (PDMS/DVB) 65 μM fiber (Supelco Analytical, Bellefonte, PA, USA) and analyzed according to the method described by González-Pérez et al. (2018). Before pouring the culture medium, each Petri dish was perforated with a sterile drill (2.0 mm diameter) to allow the SPME fiber entrance; three plates were analyzed for each condition. In each Petri dish, seven-day-old seedlings grown in an experiment performed with an indirect treatment design were placed on the side where the hole was made, and on the opposite side *H. opuntiae* was present. Next, the dishes were sealed with Parafilm® to prevent the escape of VOCs and incubated vertically for seven days at 22 ± 2 ºC in a growth chamber with a 16-h light photoperiod as described. SPME fiber was introduced into the hole and exposed for 60 min. The fiber was inserted into the injection port and desorbed for 20 min in a splitless injector at 200 °C of the gas chromatograph GC-7890b (Agilent Technologies, Santa Clara, CA, USA) coupled to a mass spectrophotometer EM-5977A (Agilent Technologies). The column used to separate the VOCs was HP-Innowax Polyethylene glycol phase capillary GC Column (30.0 m × 0.320 mm i.d. × 0.25 μm, Agilent Technologies). The column temperature conditions were set to 40 °C for 10 min, then the temperature increased at a rate of 3 °C per minute to reach a final temperature of 180 °C and held for 10 min. Helium was used as carrier gas at a constant flux rate of 1.5 mL/min. The compounds were identified by deconvolution using the W10N11 mass spectral library (Wiley10Nist11) and based on the linear retention index values (Van den Dool 1963), which were calculated after analyzing C6 and C25 n-alkanes. For each condition, the peak area of each organic compound identified was calculated as a percentage proportional to the total peak area of all volatile organic compounds. Peaks with identity lower than 90% in respect to Wiley10Nist11 library were not considered.

### In vitro inhibitory assay and plant infection with *B. cinerea*

*B. cinerea* strain BMM (isolated initially from grape wine) was provided by Brigitte Mauch-Mani (University of Neuchatel, Switzerland). Both, *B. cinerea *in vitro growth and spore suspension preparation, were carried out as previously described by L’Haridon et al. (2011)*.* For the in vitro inhibition assay*,* we used Petri dishes prepared for indirect treatment experiments. On the right side, the medium contained potato dextrose agar media (PDA) and five μL of a *B. cinerea* spore suspension of (5 × 10^4^ spores/mL), and the left side contained MS medium and 20 µL of a suspension of 1 × 10^10^ cells/mL of *H. opuntiae*. The plates were incubated at 22 ± 2 ºC for 3 days.

Growth inhibition of *B. cinerea* was evaluated by measuring the area of the *B. cinerea* hyphae. The images were processed using the ImageJ software. The number of spores was determined by harvesting mycelium, which was resuspended in distilled water and filtered through glass wool to remove hyphae. Spores were 1:1000 diluted and then observed in a Neubauer chamber at a 20 × magnification in a Zeiss Axioskop 2 microscope.

*B. cinerea* plant infection protocol and lesion size measurement were performed as previously described by L’Haridon et al. (2011). Thus, four-week-old *Arabidopsis thaliana* plants were inoculated with a 1 × 10^10^/mL cell suspension of *H. opuntiae* next to the roots every other day for 1 week. After this time, five μL droplets of *B. cinerea* spore suspension (5 × 10^4^ spores) were applied to the fully expanded leaves. Infection symptoms were evaluated at three dpi by measuring the disease incidence and lesion size on the leaf surface. Pictures were taken at three dpi with a digital camera. ImageJ software was used to estimate the lesion size (mm^2^). All experiments were performed at least three times with similar results.

### Statistical analysis

All results are reported as mean values (± SE). Student’s *t*-test analysis (*P* < 0.05) was carried out to determine statistically significant differences between treatments. The software GraphPad Prism version 9.4.0 was used (2019, GraphPad Software, San Diego, CA, USA). All the data analyzed were obtained from three independent experiments.

## Results

### In vitro analysis of the interaction between *A. thaliana* and *H. opuntiae*

**Fig. 1:**
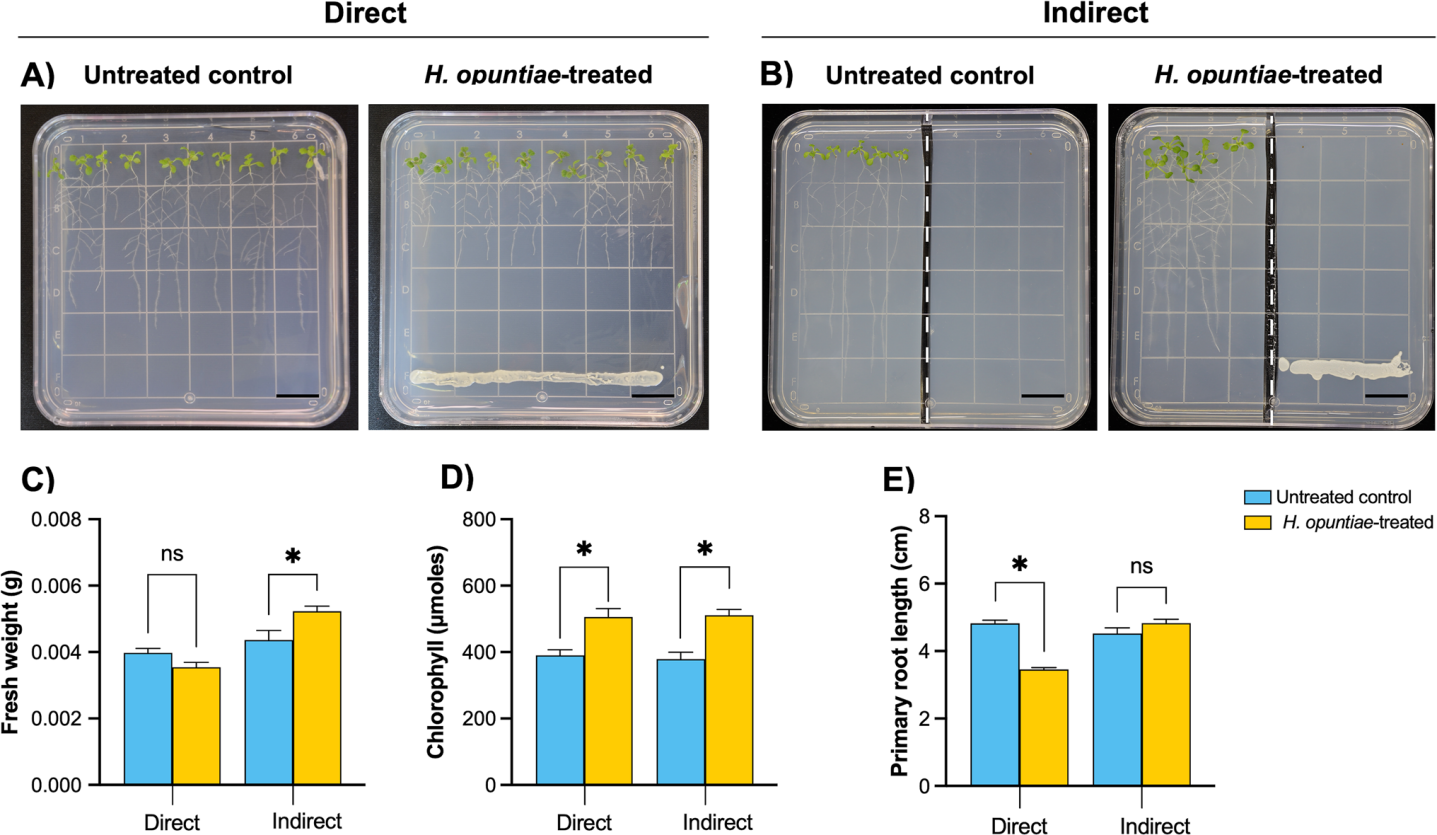
*A. thaliana* growth and root development during direct and indirect in vitro interaction systems. Representative data of 14-day-old *A. thaliana* (Col-0) plantlets grown on MS media after seven dpi with *H. opuntiae* cells. As a control, plantlets were grown on MS medium without yeast. **A**, **B** Seedlings were exposed to yeast using **A** the direct interaction system (**A**) or the indirect interaction system without direct contact (**B**). In both cases, the yeast cells were distributed on the MS medium. The scale bar corresponds to 1 cm. **C**-**E** Physiological parameters were examined in shoots and roots. **C** Fresh weight per plant (g). **D** Total chlorophyll content (μMoles). **E** Primary root length (cm). Bar plots represent mean ± SE values of dependent variables (*n* = 30). Asterisks indicate a statistically significant difference between uninoculated and inoculated samples, according to the Student’s *t*-test (*P* ≤ 0.05)

To investigate the effect of *H. opuntiae* on *A. thaliana* growth and development, we grew wild-type *A. thaliana* seedlings under normal conditions in the MS growth medium for seven days and then co-cultivated with *H. opuntiae*. After seven dpi, we recorded plant physiological parameters, such as fresh weight, chlorophyll content, and primary root growth (Fig. 1). These analyses were carried out using two different experimental conditions: a direct treatment (no physical barrier between the plants and yeast cells when the yeast culture was applied on the same agar medium surface where seedlings grew) and an indirect treatment (physical barrier: the ∼2-mm-thick agar block was removed from the middle of the plate permitting plant growth on one side of Petri dish and yeast cells on another side) (Fig. 1A, B).

Under direct treatment conditions, no plant fresh weight differences were detected when plantlets grew on a medium with *H. opuntiae* compared to those untreated control plantlets (Fig. 1C). In contrast, *H. opuntiae* indirect treatment increased the fresh weight of *A. thaliana* plantlets. Moreover, both types of treatment caused increased chlorophyll levels compared to the untreated control (Fig. 1D). Interestingly, we noticed a drastic alteration of root architecture in *A. thaliana* plants exposed to *H. opuntiae,* compared to untreated plantlets. Under direct treatment, the primary root length was significantly reduced (Fig. 1E) and was ~ 70% of that of the untreated control plants. In detail, we observed that the primary root growth started to delay after four days of *H. opuntiae* presence (Suppl. Fig. S1). In contrast, the primary root length was not different in the indirect treatment with *H. opuntiae* compared to the untreated control (Fig. 1E)*.*

**Fig. 2:**
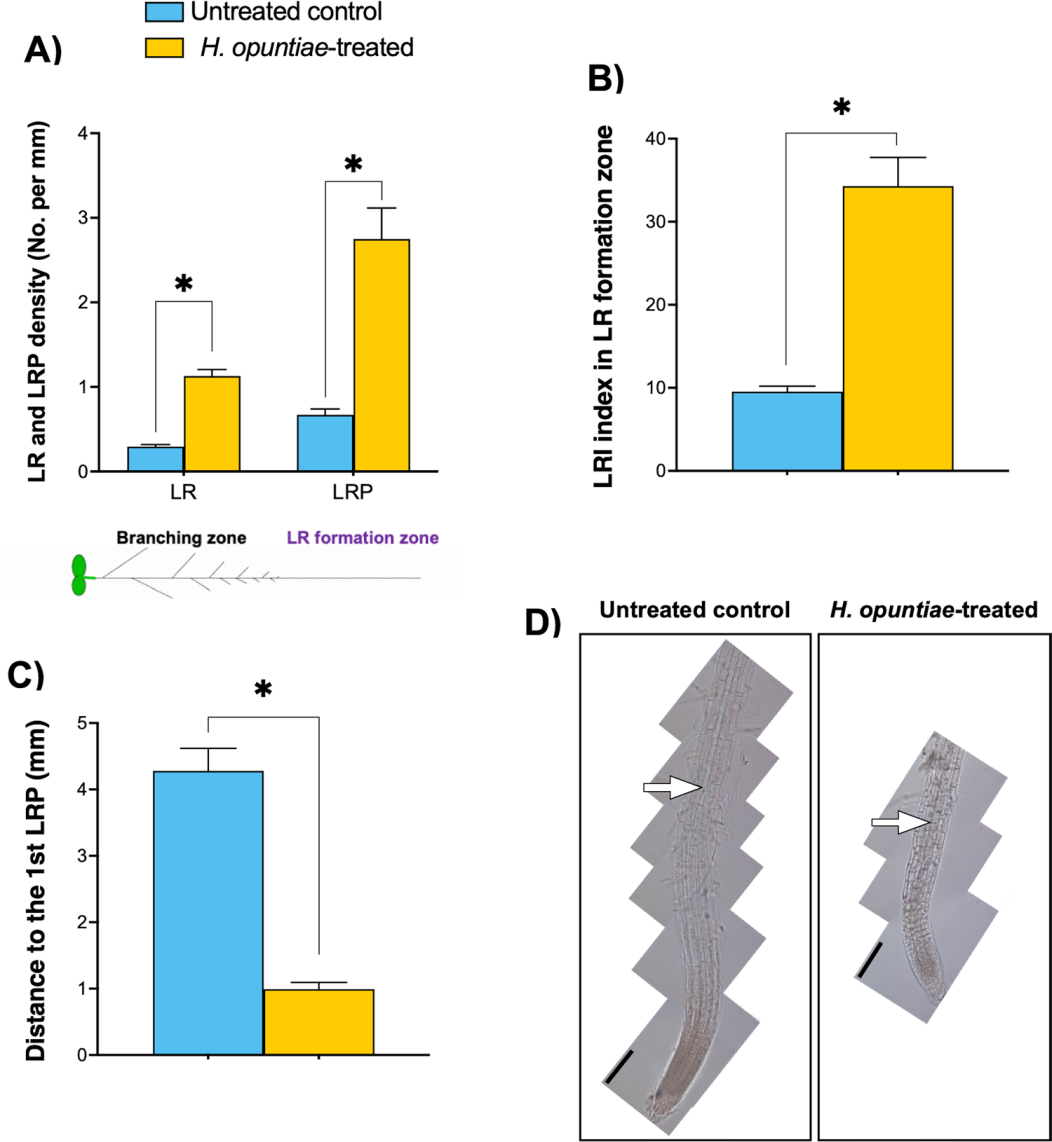
*H. opuntiae* affected the lateral root initiation in *A. thaliana* when plants are treated using in direct interaction system. **A** The lateral root (LR) density and LR primordium (LRP) density in the branching and LR formation zone. **B** The lateral root initiation index (LRI) estimates the number of LR initiation events in 100 cells. **C** Distance to the first initiated LRP in millimeters. **D** Representative pictures of Arabidopsis (Col-0) roots of untreated control (left) and *H. opuntiae* treated (right). White arrows indicate the position of the most distal differentiated xylem, which is much closer to the tip in the treated seedling (*n* = 18 and 20, respectively). The reference bar = 200 μm. Asterisks indicate a statistically significant difference between untreated control and *H. opuntiae*-treated samples, according to the Student’s *t*-test (*P* ≤ 0.05)

Since the most severe effect of yeast cells was observed during direct treatment, we selected it to explore the role of *H. opuntiae* in root development by analyzing the LR and LRP density and addressing whether LR initiation was affected (Fig. 2) (Malamy and Benfey 1997; Dubrovsky et al. 1994). We found that the density of LRs in a branching zone and the density of LRPs in the lateral root formation zone (see Fig. 2A bottom; Dubrovsky and Forde 2012) were greater in *A. thaliana* plantlets grown in a Petri dish with yeast cells in the direct experimental setup (Fig. 2A). These results suggested that in this setup, yeast promoted LR initiation (LRI) in *A. thaliana* primary root. This effect, however, can be a result of a decreased fully elongated cell length (Suppl. Fig. S1). To normalize the density data, we estimated the LR initiation index (*I*_*LRI*_), a parameter that estimates the number of LR initiation events per root portion comprising 100 cortical cells in a cell file (Dubrovsky et al. 1994). Indeed, it was found that the *I*_*LRI*_ was 3.6 times higher in treated compared to untreated seedlings (Fig. 2B), confirming that LR initiation was indeed strongly increased by the yeast. This result suggested the auxin-like effects of the yeast cells. Commonly, auxin inhibits the primary root growth, which was also affected in this experiment (Fig. 1E). This effect, in turn, is often accompanied by a decrease in distance from the root tip to the first LR initiation event. We found that in Col-0 roots treated with *H. opuntiae*, this distance was also significantly decreased (Fig. 2C*)*. Moreover, the xylem in the Col-0 treated seedling was much closer to the root tip compared to the untreated seedlings (Fig. 2D). Together these results suggest an auxin-like effect of metabolites emitted from yeast H. *opuntiae* cells over the root system architecture in *Arabidopsis*.

### Auxin and ethylene responses are induced in *A. thaliana* during the *H. opuntiae* interaction

**Fig. 3:**
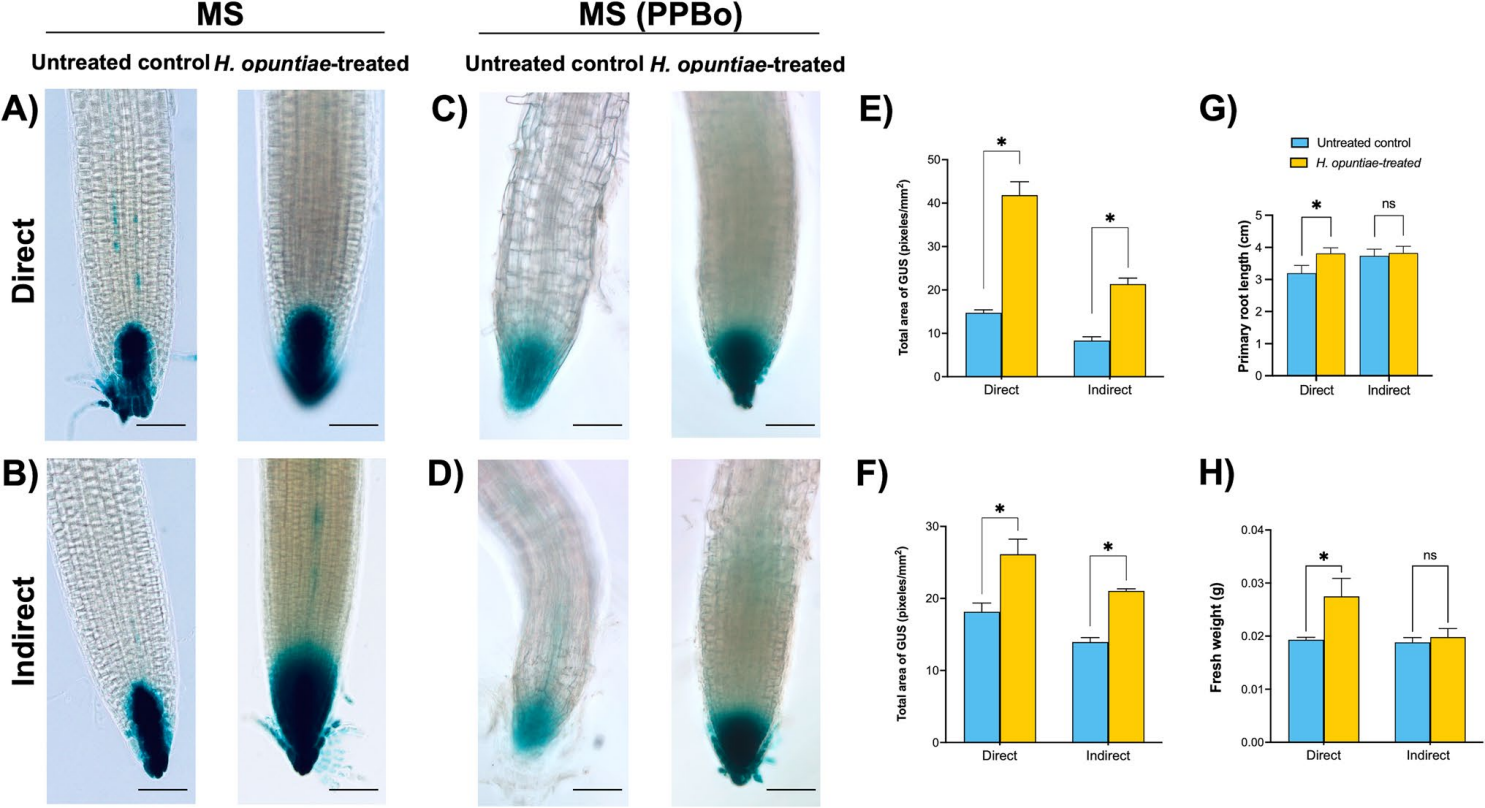
The *DR5:uidA* GUS signal increases under *H. opuntiae* interaction. Histochemical GUS staining patterns of *DR5::uidA* inoculated with *H. opuntiae* in direct (**A**) and indirect (**B**) interaction systems. A control seedling grown on MS without any inoculation was used. **C**, **D** Pharmacological inhibition of auxin biosynthesis. Effect of 4-phenoxyphenylboronic acid (PPBo, 10 μM) during the interaction between *DR5::uidA* and *H. opuntiae* in direct (**C**) and indirect (**D**) interaction systems. Pictures represent individuals from at least 15 stained GUS seedlings for each of the interaction systems. The reference bar = 75 μm. Images of primary root tips were acquired in Zeiss Axioskop 2 microscope at 10 × magnification. **E, F** GUS intensity quantification (pixels/mm2) from the primary root tip in (**A** to **D)** treatments. **G** *tir1* primary root length (cm). **H** *tir1* fresh weight per plant (g). Bar plots represent mean ± SE values of dependent variables (*n* = 30). Asterisks indicate a statistically significant difference between uninoculated and inoculated samples, according to the Student’s *t*-test (*P* ≤ 0.05)

To further investigate the role of *H. opuntiae* in root development, we examined auxin response at a transcriptional level in *A. thaliana* roots using the *DR5:uidA* reporter line (Ulmasov et al. 1997) (Fig. 3). Seven-day-old *DR5:uidA* seedlings grown in Petri dishes containing *H. opuntiae* (at both direct and indirect treatments) showed an increase in the GUS signal in the primary root tips after seven dpi (Fig. 3A, B). Notably, in relative terms, a greater auxin response was detected after indirect treatment, which was observed with a stronger GUS signal and a greater tissue volume showing GUS activity (Fig. 3B). To confirm the effect of auxin-induced response, we applied the auxin biosynthesis inhibitor, 4-phenoxyphenylboronic acid (PPBo) (Kakei et al. 2015) to plantlets treated with the yeast (Fig. 3C, D). Accordingly, pixel intensity quantification analyses showed an increase in the GUS signal intensity in the primary root tips in the presence of *H. opuntiae* (Fig. 3E, F). We found that PPBo applied during plant and yeast interaction for seven days caused a decrease in the GUS signal of *DR5:uidA* seedlings which was reverted when treated with *H. opuntiae* irrespective of the type of treatment. An increased auxin response in plants treated in both ways with *H. opuntiae* suggested that either auxin accumulation, or local auxin synthesis, or both are enhanced in plants exposed to the yeast. Also, the auxin-sensing receptor mutant *tir1* showed an increment in primary root length and fresh weight during the direct interaction conditions, while there were no differences in this parameter during indirect interaction (Fig. 3G, H), supporting the hypothesis that *H. opuntiae* direct interaction can lead to plant auxin-related responses.

**Fig. 4:**
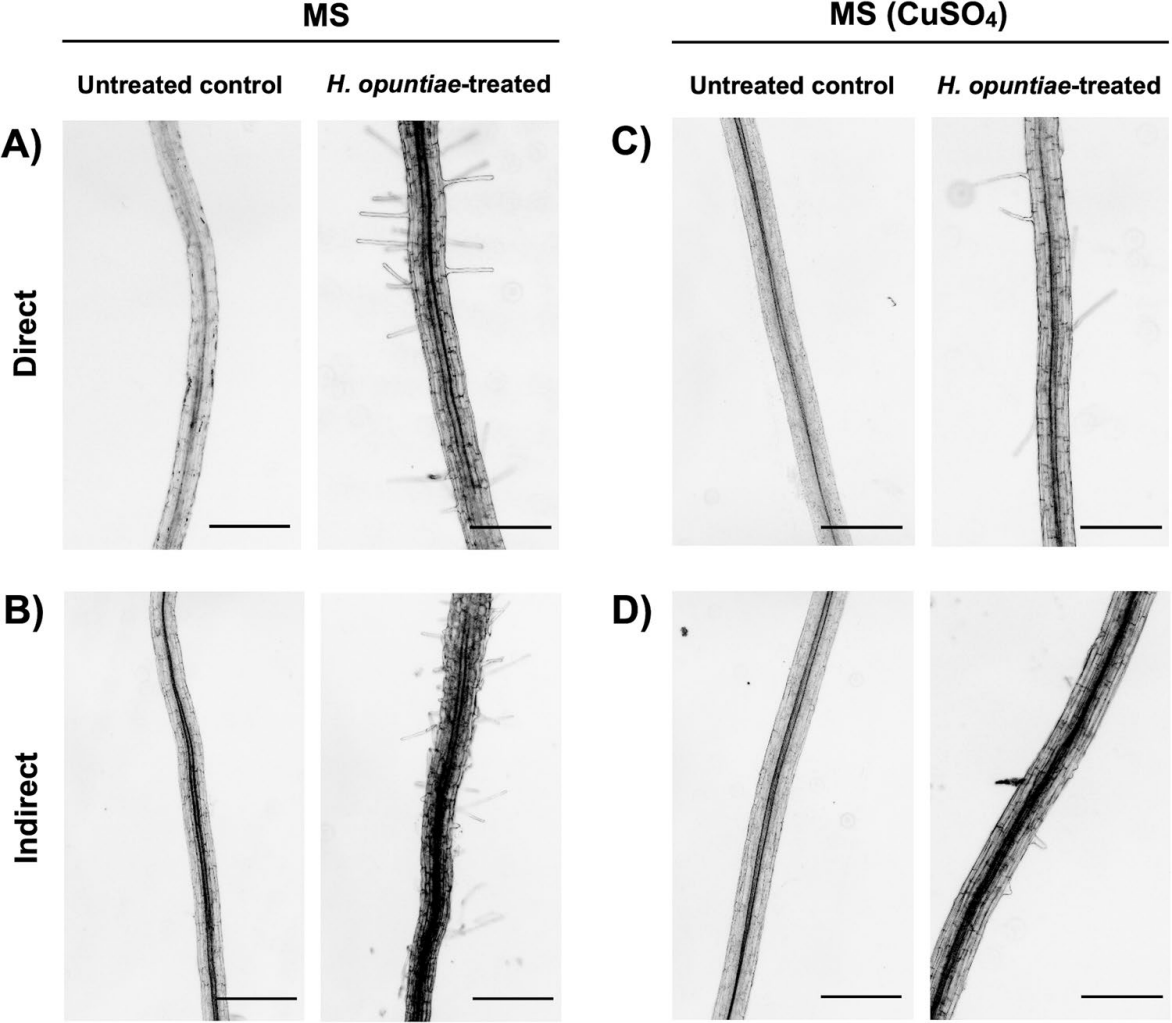
Effect of *H. opuntiae* in the *rhd6* root hair defective mutant. Seven-day-old *rhd6* mutant seedlings were inoculated with *H. opuntiae* cells for seven days under direct (**A**) and indirect (**B**) interaction systems. As a control, plantlets grew on MS without yeast application. **C**, **D** Pharmacological inhibition of ethylene signaling. Effect of CuSO_4_ (50 mM) during the interaction between *H. opuntiae* and *rhd6* mutant under direct (**C**) and indirect (**D**) interaction systems. Images were taken under a microscope (Zeiss Axioskop 2 microscope) and represented individuals from at least 15 seedlings. Root hair images were captured approximately between 500 and 1000 μm from the primary root tip. The scale bar corresponds to 500 µm

Root hairs are epidermal cell extensions that play a crucial role during nutrient acquisition, root anchorage, and environmental interactions (Vissenberg et al. 2020). It is known that, besides auxin, the phytohormone ethylene regulates root hair growth and development (Feng et al. 2017). Further, we analyzed the ethylene-associated process of root hair formation using a mutant affected in a bHLH transcription factor, ROOT HAIR DEFECTIVE 6. The *rhd6* loss-of-function mutant shows a root hair impaired phenotype under normal growth conditions (Masucci et al. 1996). RHD6 protein activates another bHLH member, *RHD6-LIKE 4* (*RSL4*), required for root hair elongation (Yi et al. 2010; Pires et al. 2013; Feng et al. 2017). When seven-day-old *rhd6* mutant seedlings were inoculated with *H. opuntiae* yeast (both direct and indirect treatments), after seven dpi we observed that the root hair defective phenotype of the *rhd6* mutant was partially reverted (Fig. 4A, B). By contrast, using the ethylene production and perception inhibitor (CuSO_4_) slightly delayed root hair growth in *A. thaliana* seedlings during both types of treatments with *H. opuntiae* cells (Fig. 4C, D). These data collectively suggest that during direct and indirect treatments, some diffused metabolites and some volatile compounds, respectively, emanate from *H. opuntiae* yeast and activate auxin- and ethylene-related responses in *A. thaliana* roots.

### Identification of Volatile Organic Compounds (VOCs) produced by *H. opuntiae*

Since we established changes in plantlet growth even without direct contact with the yeast, we aimed to identify VOCs produced by *H. opuntiae* cells. The VOCs were collected using a solid phase micro-extraction (SPME) for 1 h and analyzed by chromatography-mass spectrophotometry (GC/MS). We analyzed the VOC mixtures produced during *A. thaliana–H. opuntiae* interactions and identified six VOCs, specifically five alcohols and one aldehyde (Table 1). Notably, we identified that the most abundant VOC was 3-methyl-1-butanol, followed by ethanol in the VOCs blends (Table 1). The VOC mixtures produced by *H. opuntiae* when it was growing alone on the growth medium were also determined.

**Table 1:** Volatile compounds emitted by *H. opuntiae* when grown in the split Petri dishes with *A. thaliana* on MS growth medium (indirect growth condition)

No.	Class	Compound	Linear Retention Index	Normalized amount of volatile compound (%)
1	Alcohol	Ethanol	628	22.76 ± 7.8
2	Aldehyde	Heptaldehyde	684	0.92 ± 0.2
3	Alcohol	2-Methyl-1-propanol	724	2.77 ± 0.5
4	Alcohol	3-Methyl-1-butanol	919	72.66 ± 8.1
5	Alcohol	Hexyl alcohol	1188	0.62 ± 0.1
6	Alcohol	1-Octanol	1483	0.24 ± 0.1

The first column indicates the number of independent experiments; quantifications: mean values ± SE (*n* = 3)

When we analyzed the VOC profiles emitted by *H. opuntiae* in the absence of the plant, we identified ten volatile compounds produced by *H. opuntiae* (Suppl. Table S1). The VOC mixtures encompassed mainly alcohols, among other compounds, such as acids and ketones. As observed in the split *A. thaliana*–*H. opuntiae* interaction (Table 1), the most abundant VOCs observed were 3-methyl-1-butanol and ethanol when the yeast was grown alone (Table 1). Therefore, we hypothesize that these compounds emitted by the yeast may be primarily responsible for triggering the growth promotion of *A. thaliana*.

## *H. opuntiae *inhibits *B. cinerea* growth under in vitro conditions

**Fig. 5:**
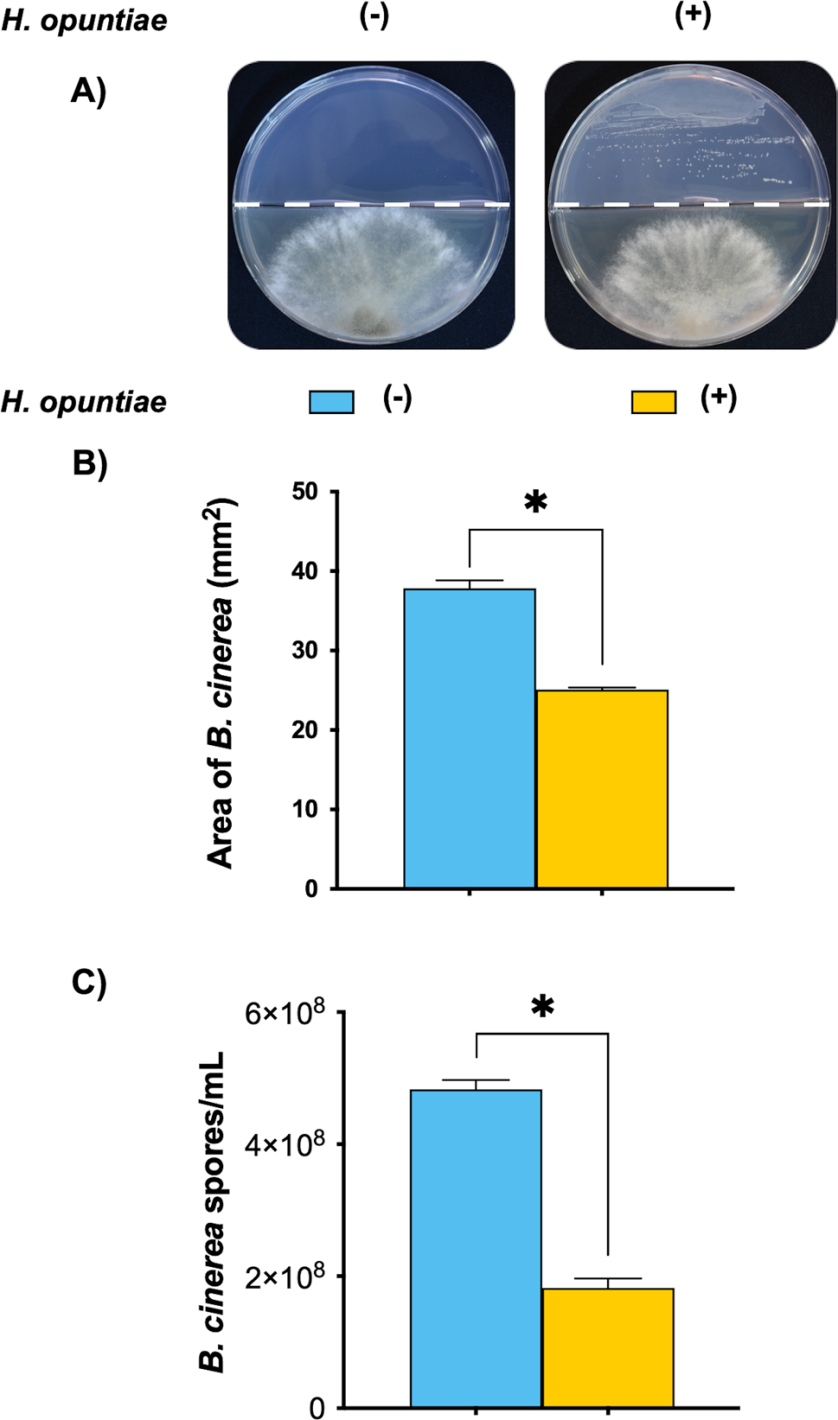
*H. opuntiae* inhibits *B. cinerea* growth in vitro. **A** *H. opuntiae* cells (6 × 10^10^ cells mL^−1^) were placed on the upper part of a split Petri dish containing MS, while on the bottom of the Petri dish, *B. cinerea* spore suspension (5 × 10^4^spores mL^−1^) was placed under PDA media. Plates were incubated at 22°C for 72 h. **B** Growth inhibition was evaluated by measuring the diameter of the *B. cinerea* mycelium. **C** Quantification of *B. cinerea* spores per mL. Bars represent the mean values (± SE) of three independent experiments. Asterisks indicate a statistically significant difference between uninoculated and inoculated samples, according to the Student's t-test (*P* ≤ 0.05)

Previously we determined that compounds released by *H. opuntiae* confer resistance to necrotrophic fungi *B. cinerea* (Ferreira-Saab et al. 2018). To analyze the potential role of *H. opuntiae* cells as a biocontrol agent, first, we determined whether *H. opuntiae* could inhibit the development of *B. cinerea* under in vitro conditions (Fig. 5A). We observed that after seven days under indirect interaction, *B. cinerea* inhibited its mycelial growth and decreased the number of spores in the presence of yeast cells (Fig. 5B, C). These results suggest that *H. opuntiae* has antifungal activity against *B. cinerea.*

### *H. opuntiae* induces plant resistance to *B. cinerea* independently of auxin, ethylene, salicylic acid, and jasmonic acid hormonal pathways

**Fig. 6:**
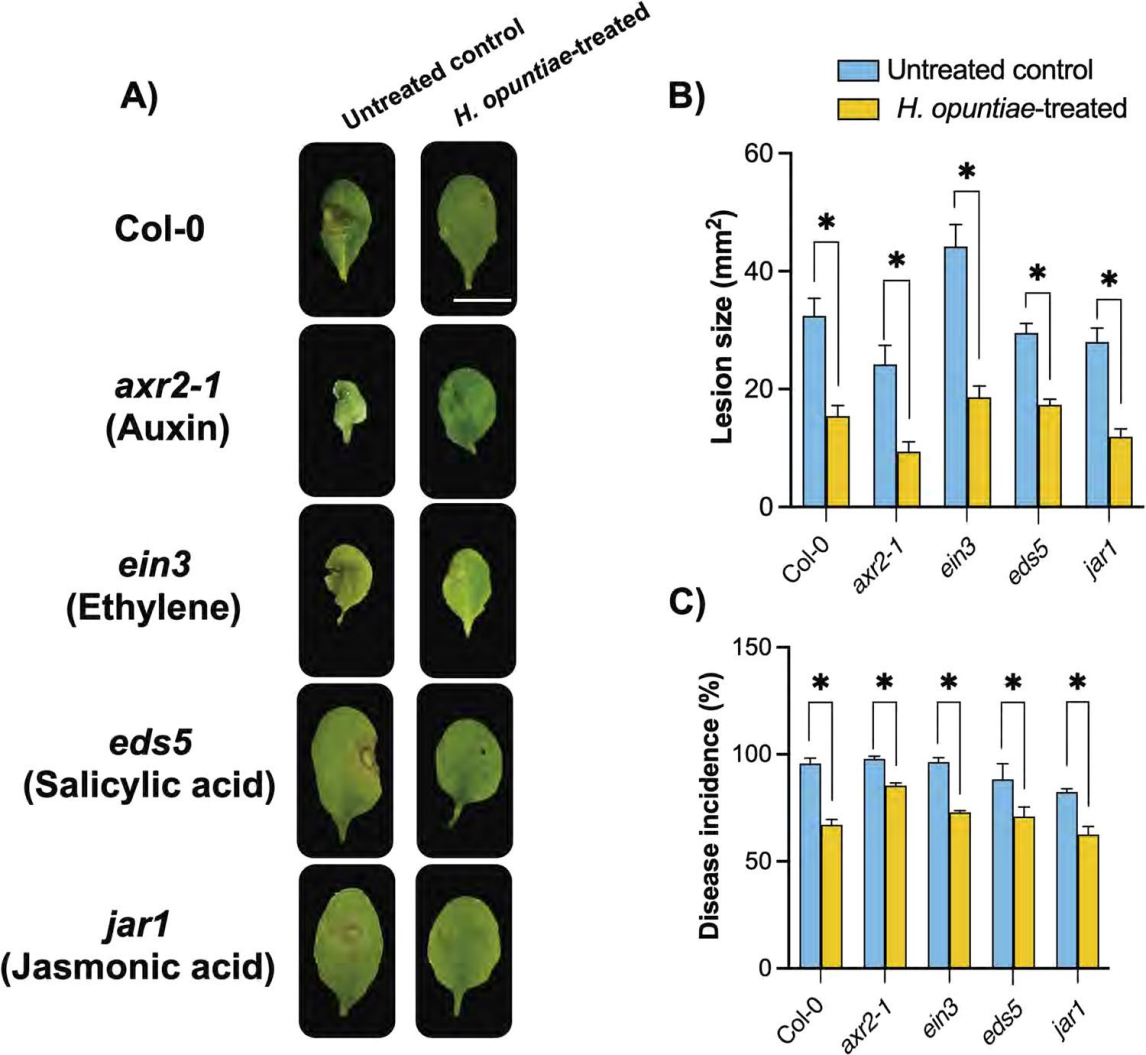
*H. opuntiae* protects *A. thaliana* plants against *B. cinerea* infection. Four-week-old *A. thaliana* plants were inoculated with MS liquid medium (Mock) or *H. opuntiae* cells (6 × 10^10^ cells mL^−1^) for 2 weeks. **A** Four-week-old *A. thaliana* plants treated with *H. opuntiae* cells were infected with five μL droplets containing a *B. cinerea* spore suspension (5 × 10^4^spores mL^−1^), and infection symptoms were evaluated at 72 hpi. Representative images of the inhibitory assay are shown. **B** Lesion size and **C** disease incidence was evaluated at 72 hpi by measuring the percentage of leaves infected per plant. Bars represent mean values (± SE) of three independent experiments, each with twenty replicates. Asterisks indicate a statistically significant difference between uninoculated and inoculated samples, according to the Student's t-test (*P* ≤ 0.05)

Previously we determined that HoFs can induce systemic acquired resistance (SAR) against *B. cinerea* (Ferreira-Saab et al. 2018). To determine if similar protection can be achieved using *H. opuntiae* cells, four-week-old wild-type *A. thaliana* plants were inoculated with yeast cell suspension every other day for one week. Next, plants were drop inoculated with a five μl of *B. cinerea* spore suspension (5 × 10^4^ spores) or water (Mock) (Fig. 6). Three dpi *A. thaliana* leaves inoculated with yeast cells showed disease resistance (Fig. 6A), as indicated by ~ 40% inhibition of disease incidence on *H. opuntiae*-treated plants compared to mock-treated ones (Fig. 6C). Moreover, the lesion size triggered by this pathogen was 50% reduced in yeast-inoculated plants than in non-inoculated plants (Fig. 6B), suggesting that *H. opuntiae* produced a protective effect in *A. thaliana* against *B. cinerea* pathogen infection.

Plant hormones, including auxin, ethylene, salicylic acid, and jasmonic acid, play a central role in regulating plant defense responses. Since we determined that *H. opuntiae* cells can induce auxin- and ethylene-related responses (Figs. 3 and 4), we addressed whether *H. opuntiae* may regulate the plant resistance to *B. cinerea* through these phytohormone-mediated pathways. We explored if *H. opuntiae* also stimulated plant defense responses by salicylic acid and jasmonic acid. For this purpose, we analyzed the fungal disease incidence and lesion size in *A. thaliana* mutant plants impaired in auxin (*axr2-1*), ethylene (*ein3*), salicylic acid (*eds5, npr1-1, and npr3-1*), and jasmonic acid (*jar1*) pathways (Staswick et al. 1992; Timpte et al. 1994; Chao et al. 1997; Nawrath et al. 2002) during the interaction with yeast cells (Fig. 6).

As shown in Fig. 6A, four-week-old *axr2-1, ein3, eds5,* and *jar1* plants under mock treatment showed severe disease symptoms of *B. cinerea* three dpi, as indicated by the high number of infected leaves and larger lesion size compared with the wild type (Fig. 6B, C)*.* Interestingly, in *axr2-1, ein3, eds5,* and *jar1* plants inoculated with *H. opuntiae,* the progression of fungal infection did not differ from WT yeast-inoculated plants (Fig. 6A). These data agree with the reduction in around 25% of disease incidence and 50% decrease of lesion size in *axr2-1, ein3, eds5,* and *jar1* yeast-inoculated compared to non-inoculated plants (Fig. 6B, C). Lastly, *npr1-1* and *npr3-1* disclosed similar reductions in lesion size in the presence of *H. opuntiae* (Suppl. Fig. S2). These data suggest that *H. opuntiae* interaction can lead to plant resistance to *B. cinerea* independently of auxin, ethylene, salicylic acid, and jasmonic acid hormonal pathways.

## Discussion

In nature, plants interact with diverse communities of microorganisms (Wang et al. 2016; Khare et al. 2018), establishing beneficial interactions, which outcome in a boost in plant growth and improving resistance and tolerance against biotic and abiotic stresses (Jones et al. 2019; Khan et al. 2021). The present study examined the interaction between *A. thaliana* and *H. opuntiae,* a fermentative yeast found on fruit surfaces (Ferreira-Saab et al. 2018; Hu et al. 2020; Saubin et al. 2020). In vitro experiments showed that *H. opuntiae* increased fresh weight and total chlorophyll content on *A. thaliana* plantlets treated in indirect mode. We established that the yeast causes substantial changes in *A. thaliana* root development resulting in shorter but more branched roots; these root architecture modifications resembled those triggered by auxin. In line with this, auxin reporter lines and pharmacological inhibition of auxin synthesis confirmed auxin-like effects. Moreover, these findings are supported by a previous report where *H. opuntiae* cells yeast exhibited the ability to produce indole-3-acetic acid (IAA) (Peng et al. 2018); however, *H. opuntiae* indirectly may affect plant growth by competing for both space and nutrients, since during direct interaction Arabidopsis fresh weight is reduced.

Auxin is central to plant and microorganism development and growth processes (Kunkel and Harper 2018). In plants, auxin is an important developmental regulator involved in cell division and elongation, differentiation, tropisms, apical dominance, senescence, abscission, and flowering (Woodward and Bartel 2005; Teale et al. 2006). Auxin production by a microorganism can function as a signaling molecule regulating microbial gene expression, promoting growth-form switching and potentially as a quorum-sensing molecule (Rao et al. 2010). In this respect, it is highly possible that an inhibitory effect of *H. opuntiae* in direct treatment experiments was caused by the auxin produced by the yeast cells, explaining the absence of a similar effect after the indirect treatment (Fig. 1E).

Pioneer reports have shown that microorganisms can regulate plant development and growth, such as in the case of *Trichoderma virens*, which increased biomass and promoted lateral root development in *A. thaliana* seedlings in an auxin-dependent manner (Contreras-Cornejo et al. 2009). In another report, González-Pérez et al. (2018) observed that the direct contact interaction between *A. thaliana* seedlings and *Trichoderma* spp abated the GUS signal in the primary root tips of the *DR5:uidA* reporter line. The authors suggest that fungi release secondary metabolites, decreasing the auxin accumulation, and thereby affecting the primary root growth. In contrast, we observed that the GUS signal of *DR5:uidA* was increased during direct interaction between *A. thaliana* and *H. opuntiae*, suggesting that microbe-produced auxin is functional, similar to one produced by plants, and produces a typical auxin response at a transcriptional level. For the moment, it is unclear how to explain an increased auxin response found after an indirect treatment with *H. opuntiae* (Fig. 3B, D), as diffusion of hypothetically produced auxins is not possible in this experimental setup. One possibility is that ethylene that can be produced by yeast cells (Thomas and Spencer 1978) induces auxin responses as auxin-ethylene cross-talk is well documented in *A. thaliana* and Trp-dependent auxin biosynthesis is upregulated by ethylene (Stepanova et al. 2005, 2007; Ivanchenko et al. 2008). However, the inhibition of root growth might indicate a hypersensitive response to diffusible compounds produced by yeast.

We observed an increase in the primary root length and fresh weight of *tir1* mutant plants when they were exposed to *H. opuntiae* in direct interaction conditions (Fig. 3G, H), contrary to what is observed in the wild-type plants. In Arabidopsis, the F-box TIR1/AFB family of proteins that act as auxin receptors regulating the induction of the downstream nuclear signaling pathway leading to the transcriptional plant reprogramming (Watanabe et al. 2017; Die et al. 2018; Leyser 2018). It has been reported that the formation of the TIR1/AFB–Aux/IAA complex is required for root growth regulation by an independent transcriptional branch of this signaling pathway (Fendrych et al. 2018). Likely, the absence of TIR1 could lead to no effect or response during auxin application, in this sense, the primary root length and fresh weight increment in the *tir1* mutant can be related to an inefficient auxin perception.

Along with ethylene, auxins are fundamental hormones controlling plant growth and development (Poupin et al. 2016). We found that *H. opuntiae* could restore the root hair defective phenotype of the *rhd6* mutant background, which is re-established by exogenous application of auxin or ethylene (Masucci et al. 1996). Additionally, in many aspects of plant immunity and disease resistance, auxin and ethylene are regulator molecules (Llorente et al. 2008; Zhao et al. 2012; Song et al. 2014; Guan et al. 2015; Kunkel and Harper 2018).

The microorganisms produce a wide range of secondary metabolites with biological activity, including VOCs. When plants perceive microbial VOCs, they can trigger a broad-spectrum response, including plant growth promotion or inhibition, and induce plant resistance against biotic and abiotic stresses (Kanchiswamy et al. 2015; Jalali et al. 2017; Garbeva and Weisskopf 2020; Zhang et al. 2020; Ruangwong et al. 2021). The VOC emission depends on the genus and species of the microorganism as well as environmental factors such as temperature, pH, availability of nutrients, and oxygen levels (Insam and Seewald 2010; Hung et al. 2015; González-Pérez et al. 2022). Alcohols have broad effects on plants (Piechulla et al. 2017), and they represent around 16% of the VOCs produced by microorganisms (Schenkel et al. 2015). In this study, we found that during the *A. thaliana-H. opuntiae* interaction the VOCs identified were mainly alcohols of which the most abundant were 3-methyl-1-butanol, ethanol, and 2-methyl-1-propanol. Regarding ethanol, this compound has been reported in VOCs blends emitted by Bacillus and Trichoderma species (Farag et al. 2006; Lee et al. 2016; Wonglom et al. 2020; Elsherbiny et al. 2020). With respect to 3-methyl-1-butanol this compound also has been detected in VOCs emitted by different microorganisms which have been reported to promote plant growth (Ledger et al. 2016; Lee et al. 2016; Heenan-Daly et al. 2021; Kong et al. 2021). In this way, 3-methyl-1-butanol significantly increased fresh weight and total chlorophyll after exposure for 72 h to 3-methyl-1-butanol at 10 ng/L in *A. thaliana* plantlets (Lee et al. 2019). Also, seeds of maize and wheat treated with a concentration of 1 and 10 mg/L^−1^ of 3-methyl-1-butanol increase germination rate and seedling growth (Li et al. 2018). Evidence reported by Kong et al. (2021) and Ledger et al. (2016) shows that 3-methyl-1-butanol improves plant growth of *Medicago sativa* and *A. thaliana* under iron deficiency and salt stress.

The branched alcohol, 2-methyl-1-propanol, has been identified in VOCs blends emitted by beneficial plant-associated microorganism’s such as Bacillus, Pseudomonas, Phoma, and Trichoderma (Farag et al. 2006; Xie et al. 2009; Naznin et al. 2013; Park et al. 2015; Lee et al. 2016). The application of 2-methyl-1-propanol showed a positive effect on plant growth promotion in Arabidopsis plantlets (Lee et al. 2019). In this way, 2-methyl-propanol was found as main component in the volatile blends produced by *Phoma* sp. (GS8-3 strain). Tobacco plants exposed to of 2-methyl-propanol mixed with 3-methyl-1-butanol and other compounds identified showed a significant increase in the fresh weight over control condition (Naznin et al. 2013). The emission of hexyl alcohol (1-hexanol) by different rhizosphere bacteria has been reported, when Arabidopsis plantlets were exposed to this compound that showed a weak plant growth promotion (Blom et al. 2011). Additionally, was reported that fungal VOCs have been implicated in regulating *A. thaliana* root development and plant growth through auxin transport and signaling (González-Pérez et al. 2018; Li et al. 2022). In this study, we found that 3-methyl-1-butanol was the dominant compound in the VOC mixtures during *A. thaliana*–*H. opuntiae* interactions. Is probable that VOCs work synergistically with the other compounds here identified playing a role in growth promotion on *A. thaliana* when interact with *H. opuntiae.*

Afterward, we analyzed the resistance to *B. cinerea* of plants root inoculated with *H. opuntiae*. Previously, it was found that compounds released by the *H. opuntiae* (HoFs) conferred systemic protection in *A. thaliana* plants against *B. cinerea* infection (Ferreira-Saab et al. 2018). We cannot discard the idea that compounds with similar activity to HoFs can be released when *A. thaliana* leaves are inoculated with yeast suspension. There is also the possibility that these compounds can penetrate the leaf tissues and cells and can induce defense responses as genuine elicitors once inside the plant cell. It was already suggested that HoFs might act as elicitors inducing the pathways of jasmonic acid- and ethylene-related defense responses (Ferreira-Saab et al. 2018). Similarly, our data indicate that *H. opuntiae* generated plant defense responses at the systemic level, which might mediate defense signaling pathways. Remarkably, we determined that canonical responses induced by ethylene, salicylic acid (SA), and jasmonic acid (JA) apparently do not have a crucial participation in *H. opuntiae*-induced defense response. This opens the possibility of identifying other uncharacterized mechanisms that might be involved in the defense against *B. cinerea* induced by this yeast that are currently studied in our group.

Finally, we hypothesize that metabolites produced by *H. opuntiae* trigger plant defenses, virulence gene expression, and stress responses that limit pathogen colonization. With this in mind, *H. opuntiae* may represent a new eco-friendly alternative to synthetic fungicides. Since *H. opuntiae* is usually present during the fermentation of fruit musts, apparently, this yeast species is not associated with a harmful effect on human or animal health.

### Supplementary Information

Below is the link to the electronic supplementary material.

Supplementary Fig. S1 Root growth kinetics. The Arabidopsis primary root length (cm) measurements were recorded in A. thaliana seedlings treated with H. opuntiae and untreated control in both direct (A) and indirect (B) experimental conditions. Bar plots represent mean ± SE values of dependent variables (n = 10 and 5, respectively). C Length of fully elongated cells in µm in untreated and H. opuntiae-treated samples (n = 18 and 20, respectively). Bars represent the mean values (± SE) of three independent experiments. Asterisks indicate a statistically significant difference between uninoculated and inoculated samples, according to the Student's t-test (P ≤ 0.05)

Supplementary Fig. S2 H. opuntiae protects A. thaliana plants against B. cinerea infection. Four-week-old (Col-0, npr1-1, and npr3-1) A. thaliana plants were inoculated with MS liquid medium (Mock) or H. opuntiae cells (6x1010 cells mL−1) for two weeks. A Four-week-old A. thaliana plants treated with H. opuntiae cells were infected with five μL droplets containing a B. cinerea spore suspension (5x104spores mL−1), and infection symptoms were evaluated at 72 hpi. Representative images of the inhibitory assay are shown. B Lesion size was evaluated at 72 hpi by measuring the percentage of leaves infected per plant. Bars represent mean values (± SE) of three independent experiments, each with twenty replicates. Asterisks indicate a statistically significant difference between uninoculated and inoculated samples, according to the Student's t-test (P ≤ 0.05)

Supplementary file3 (DOCX 17 KB)

## Data Availability

Materials described in the manuscript, including all relevant raw data, will be freely available to any researcher wishing to use them for non-commercial purposes, without breaching participant confidentiality.

## Abbreviations

dpi: Days post-inoculation
HoFs: *H. opuntiae*-Filtrates
LR: Lateral root
LRP: LR primordium
VOC: Volatile organic compound
