# Supplementary figures and images for: A biostimulant yeast, Hanseniaspora opuntiae, modifies Arabidopsis thaliana root architecture and improves the plant defense response against Botrytis cinerea
Source: Planta. 2024 Jan 31;259(3):53. doi: 10.1007/s00425-023-04326-6 (PMC10830669; doi:10.1007/s00425-023-04326-6)

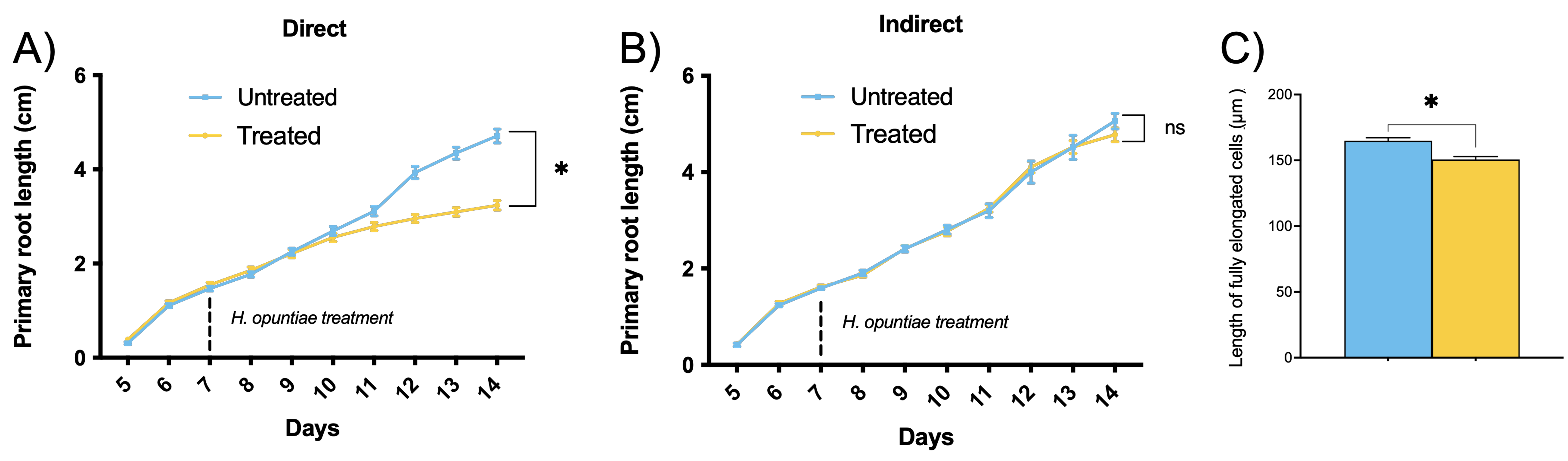

Supplement: Supplementary file 1 — Supplementary Fig. S1 Root growth kinetics. The Arabidopsis primary root length (cm) measurements were recorded in A. thaliana seedlings treated with H. opuntiae and untreated control in both direct (A) and indirect (B) experimental conditions. Bar plots represent mean ± SE values of dependent variables (n = 10 and 5, respectively). C Length of fully elongated cells in µm in untreated and H. opuntiae-treated samples (n = 18 and 20, respectively). Bars represent the mean values (± SE) of three independent experiments. Asterisks indicate a statistically significant difference between uninoculated and inoculated samples, according to the Student's t-test (P ≤ 0.05) [file 425_2023_4326_MOESM1_ESM.tiff]

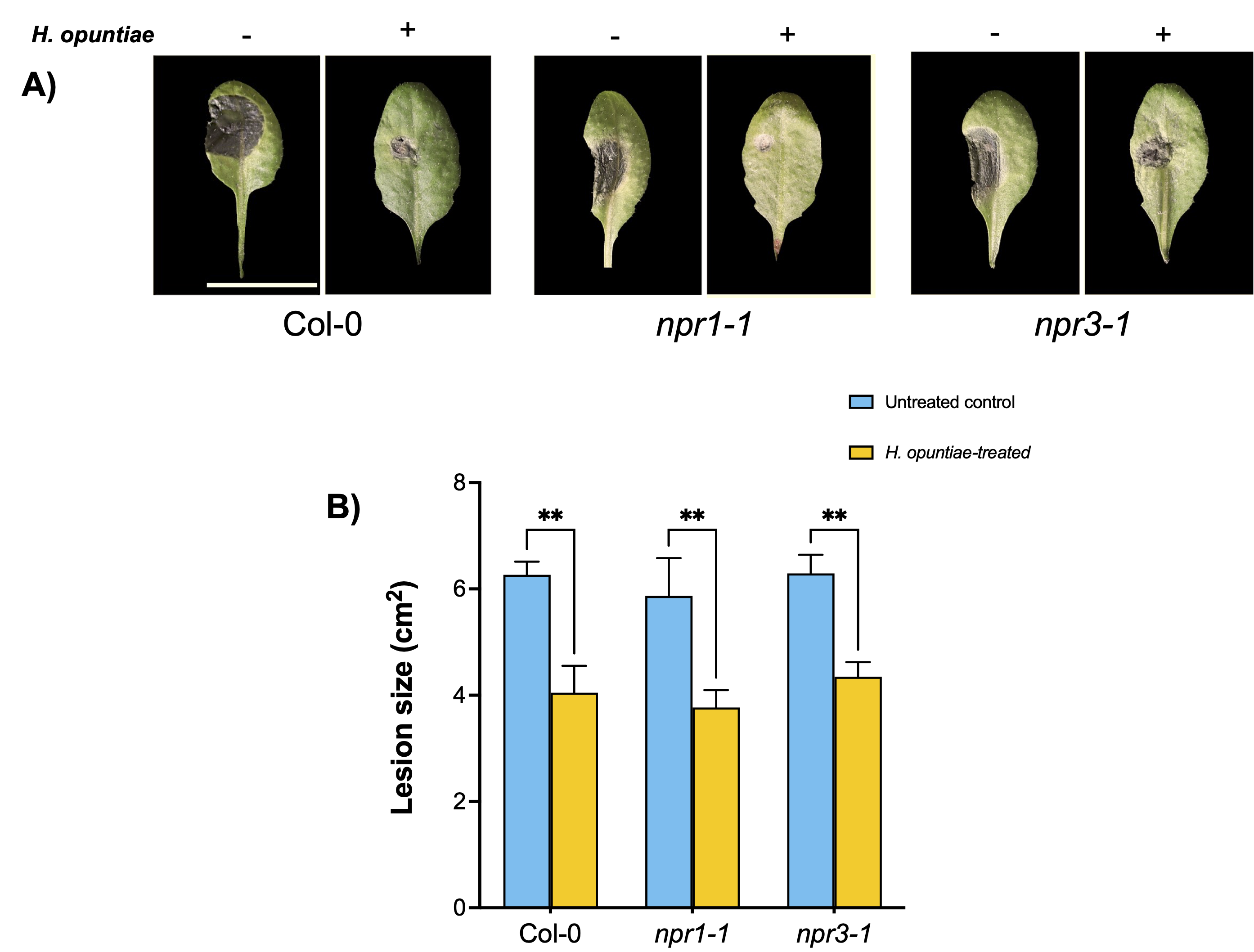

Supplement: Supplementary file 2 — Supplementary Fig. S2 H. opuntiae protects A. thaliana plants against B. cinerea infection. Four-week-old (Col-0, npr1-1, and npr3-1) A. thaliana plants were inoculated with MS liquid medium (Mock) or H. opuntiae cells (6x10^10^ cells mL−1) for two weeks. A Four-week-old A. thaliana plants treated with H. opuntiae cells were infected with five μL droplets containing a B. cinerea spore suspension (5x10^4^spores mL−1), and infection symptoms were evaluated at 72 hpi. Representative images of the inhibitory assay are shown. B Lesion size was evaluated at 72 hpi by measuring the percentage of leaves infected per plant. Bars represent mean values (± SE) of three independent experiments, each with twenty replicates. Asterisks indicate a statistically significant difference between uninoculated and inoculated samples, according to the Student's t-test (P ≤ 0.05) [file 425_2023_4326_MOESM2_ESM.tiff]
