# Supplementary material for: A biostimulant yeast, Hanseniaspora opuntiae, modifies Arabidopsis thaliana root architecture and improves the plant defense response against Botrytis cinerea
Source: Planta. 2024 Jan 31;259(3):53. doi: 10.1007/s00425-023-04326-6 (PMC10830669; doi:10.1007/s00425-023-04326-6)
Supplement: Supplementary file 3 — Supplementary file3 (DOCX 17 KB) [file 425_2023_4326_MOESM3_ESM.docx]

**Supplementary Table S1** Volatile compounds emitted by *H. opuntiae* growth on MS growth medium without *A. thaliana* plants.

| **No.** | **Class** | **Compound** | **Linear**  **Retention**  **Index** | **Normalized amount of volatile compound (%)** |  |
| --- | --- | --- | --- | --- | --- |
|  |  |  |  |  |  |
| 1 | Alcohol | Ethanol | 628 | 27.43 ± 4.3 |  |
| 2 | Alcohol | 2-Methyl-1-propanol | 724 | 3.21 ± 0.33 |  |
| 3 | Alcohol | 3-Methyl-1-butanol | 919 | 67.01 ± 4.2 |  |
| 4 | Alcohol | 2H-Pyran-3-ol | 1052 | 0.40 ± 0.1 |  |
| 5 | Alcohol | 1-Pentanol | 1144 | 0.22 ± 0.1 |  |
| 6 | Alcohol | Hexyl alcohol | 1188 | 0.44 ± 0.2 |  |
| 7 | Alcohol | 1-Heptanol | 1343 | 0.31 ± 0.1 |  |
| 8 | Ketone | 1-Phenyl-ethanone | 1579 | 0.37 ± 0.1 |  |
| 9 | Acid | 2-Pyridinepropanoic acid | 1834 | 0.33 ± 0.1 |  |
| 10 | Alcohol | Phenethyl alcohol | 1995 | 0.27 ± 0.1 |  |

The first column indicates the number of independent experiments; quantifications: mean values ± SE (*n* = 3)
